# Supplementary material for: Towards unified AI-driven fracture mechanics: the extended deep energy method (XDEM)
Source: Nat Commun. 2026 Aug 15;17:8492. doi: 10.1038/s41467-026-76748-1 (PMC13484471; doi:10.1038/s41467-026-76748-1)
Supplement: Supplementary file 3 [file 41467_2026_76748_MOESM3_ESM.zip › pic/Results/Crack_propagation/Straight/2D_contourf.pdf]

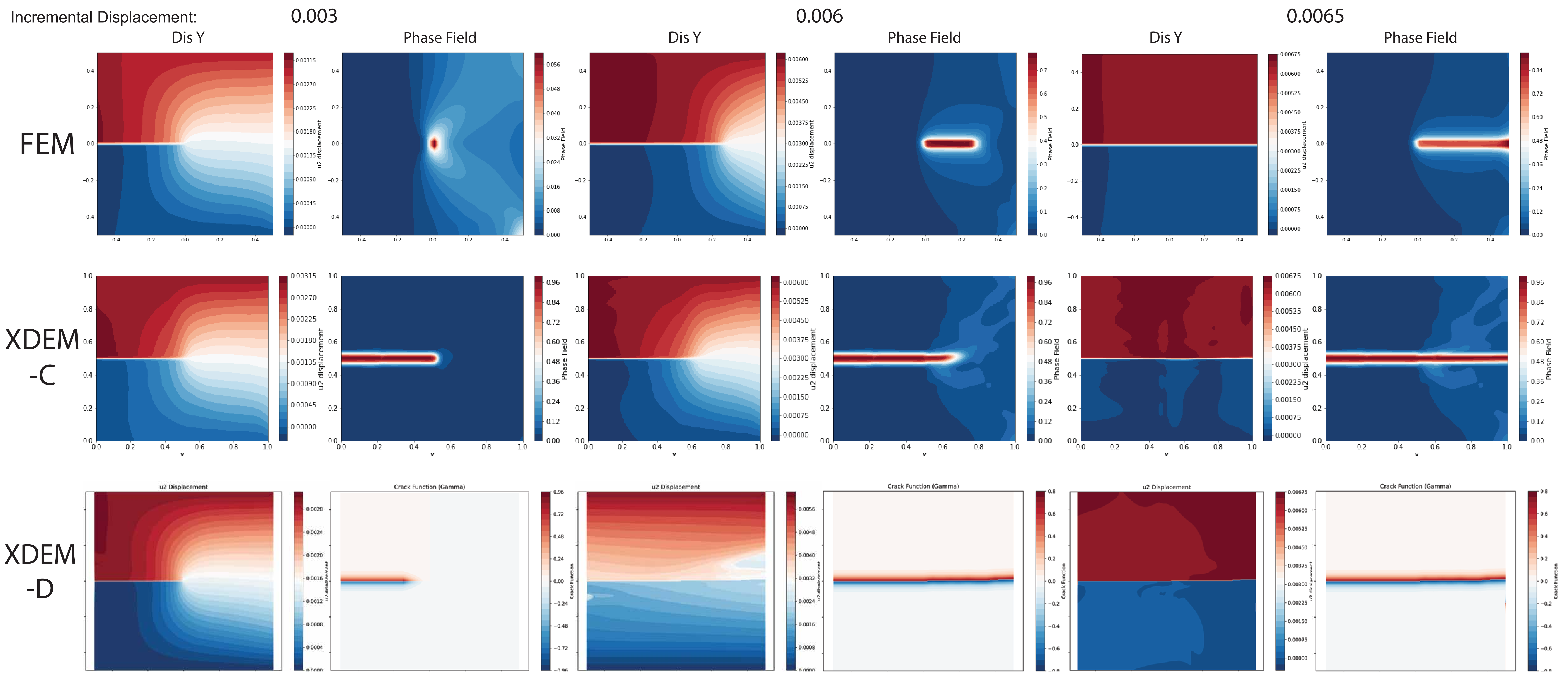

Incremental Displacement:
0.003
0.006
0.0065
Dis Y
Phase Field
FEM
XDEM -C
XDEM -D
u2 displacement
Phase Field
u2 Displacement
Crack Function (Gamma)
Crack Function
